# Supplementary material for: COZOID: contact zone identifier for visual analysis of protein-protein interactions
Source: BMC Bioinformatics. 2018 Apr 6;19:125. doi: 10.1186/s12859-018-2113-6 (PMC5889581; doi:10.1186/s12859-018-2113-6)
Supplement: Supplementary file 2 — Software build. Executable binary file of the software tool. (ZIP 118,784 kb) [file 12859_2018_2113_MOESM2_ESM.zip › cozoid/java/docs/beansbinding-1.2.1-doc.zip!/org/jdesktop/el/impl/lang/VariableMapperFactory.html]

VariableMapperFactory (Beans Binding)


---


|  |  |  |
| --- | --- | --- |
| **Overview** \| **Package** \| **Class** \| **Use** \| **Tree** \| **Deprecated** \| **Index** \| **Help** | |  |
| **PREV CLASS**   **NEXT CLASS** | **FRAMES**    **NO FRAMES**     **All Classes** |  |
| SUMMARY: NESTED \| FIELD \| CONSTR \| METHOD | DETAIL: FIELD \| CONSTR \| METHOD |  |


---


## org.jdesktop.el.impl.lang Class VariableMapperFactory

```
java.lang.Object
  org.jdesktop.el.VariableMapper
      org.jdesktop.el.impl.lang.VariableMapperFactory
```

---

``` public class VariableMapperFactory extends VariableMapper ```

---

**Constructor Summary**

`VariableMapperFactory(VariableMapper target)`


| **Method Summary** | |
| --- | --- |
| `VariableMapper` | `create()` |
| `ValueExpression` | `resolveVariable(java.lang.String variable)` |
| `ValueExpression` | `setVariable(java.lang.String variable, ValueExpression expression)`             Assign a ValueExpression to an EL variable, replacing any previously assignment to the same variable. |

**Methods inherited from class java.lang.Object**

`clone, equals, finalize, getClass, hashCode, notify, notifyAll, toString, wait, wait, wait`

**Constructor Detail**

### VariableMapperFactory

```
public VariableMapperFactory(VariableMapper target)
```


**Method Detail**

### create

```
public VariableMapper create()
```

---


### resolveVariable

```
public ValueExpression resolveVariable(java.lang.String variable)
```

:   **Specified by:**: `resolveVariable` in class `VariableMapper`
:   **Parameters:**: `variable` - The variable name **Returns:**: the ValueExpression assigned to the variable, null if there is no previous assignment to this variable.

---


### setVariable

```
public ValueExpression setVariable(java.lang.String variable,
                                   ValueExpression expression)
```

:   **Description copied from class: `VariableMapper`**
:   Assign a ValueExpression to an EL variable, replacing
    any previously assignment to the same variable.
    The assignment for the variable is removed if
    the expression is `null`.

    :   **Specified by:**: `setVariable` in class `VariableMapper`
    :   **Parameters:**: `variable` - The variable name: `expression` - The ValueExpression to be assigned to the variable. **Returns:**: The previous ValueExpression assigned to this variable, null if there is no previouse assignment to this variable.


---


|  |  |  |
| --- | --- | --- |
| **Overview** \| **Package** \| **Class** \| **Use** \| **Tree** \| **Deprecated** \| **Index** \| **Help** | |  |
| **PREV CLASS**   **NEXT CLASS** | **FRAMES**    **NO FRAMES**     **All Classes** |  |
| SUMMARY: NESTED \| FIELD \| CONSTR \| METHOD | DETAIL: FIELD \| CONSTR \| METHOD |  |


---
